# Investigation of the Possibilities for Infrared Diagnosis of Peirce–Smith Converters in Non-Ferrous Metallurgy

**DOI:** 10.3390/ma18184383

**Published:** 2025-09-19

**Authors:** Emil Mihailov, Daniela Choshnova, Maria Ivanova, Monika Asenova

**Affiliations:** Department of Physical Metallurgy and Thermal Aggregates, Faculty of Metallurgy and Material Science, University of Chemical Technology and Metallurgy, 8 Kliment Ohridski Blvd., 1756 Sofia, Bulgaria; daniela@uctm.edu (D.C.); m_ivanova@uctm.edu (M.I.); monikaji@abv.bg (M.A.)

**Keywords:** infrared diagnosis, Peirce–Smith converter, predictive maintenance, non-ferrous metallurgy, mathematical modeling

## Abstract

To implement predictive maintenance of units in the practice of metallurgical manufacturers, computer information and diagnostic systems are being developed to assess the current state of individual units throughout their entire life cycle. This publication presents the results of a study on developing an infrared diagnostic system for predictive maintenance of converter units in the non-ferrous metallurgy industry. A 3D mathematical model of the transient heat transfer in the wall of a real operating unit has been developed and numerically implemented to study, analyze, and diagnose surface temperature fields resulting from wear and local damage. To adjust the operation of the mathematical model, the design parameters and the results for operating and technological parameters from an industrial experiment are taken into consideration. Using the model, a full-factor experiment was simulated to study the surface temperature fields resulting from the erosion wear of the wall and the presence of local damage. Based on the simulation results, the optimal time range for thermographic monitoring is determined. A regression dependence was derived to predict the refractory wall wear as a function of the outer surface temperature of the converter unit. The results are part of a comprehensive investigation aimed at developing thermal imaging techniques for converter units in non-ferrous metallurgy.

## 1. Introduction

Thermal shocks and the physicochemical impact of the liquid metal on the refractory insulation, together with the continuous nature of metallurgical technologies and the intensification of production, increase the risk of damage and disruption of the structural integrity of the units during the technological process. The destruction of the structural integrity of the metallurgical furnaces and equipment results in the forced interruption of production and significant costs associated with emergency response. It requires measures to be taken to reduce the risk of such situations. An opportunity to address these issues in the metallurgical industry is the implementation of decision-making systems and predictive maintenance into the production process. They are based on continuous or periodic monitoring and diagnostics of the refractory insulation condition in high-temperature furnaces and auxiliary equipment [1,2,3].

The complexity of technological operations, the conditions under which they occur, the size of industrial equipment, and the volume of data generated by periodic measurements in combination with those of the operating parameters of the process, make subjective assessment extremely difficult and require the development of specific approaches and rules for monitoring, evaluation, and interpretation of inspection data. This is combined in the work of modern systems for diagnostics and making informed decisions based on the processing of results from various monitoring approaches [4,5]. The implementation of such systems extends the life cycle, leading to the safe operation and increased environmental, operational, and economic efficiency of technological facilities within the context of Industry 4.0. Various approaches are used to assess the condition of the refractory insulation in the units, starting with visual assessments by experts and ending with modern infrared thermography systems [6,7] and laser scanning systems for the interior of the metallurgical unit [8]. In [9], a system for monitoring the condition of refractory insulation in steelmaking units is described as part of the overall information system for production processes. Condition monitoring procedures involve processing the values of measurable parameters to identify significant changes that indicate a developing failure, forming the basis of predictive maintenance procedures. Condition monitoring [10] enables the planning of maintenance activities and taking actions to prevent failures and avoid accidents. Using the accumulated results for the parameters on which a condition assessment is based, the future behavior and remaining life of the unit are predicted [11,12,13]. In the implementation of such systems, image preprocessing operations are applied, which are necessary for recovering from degradation, camera noise, and dead pixels, and subsequently converting them into temperature values [14]. No less important are the procedures for defect detection, quantitative characterization of defects, and assessment of their severity. In [15,16], a system for detecting hot spots on the surface of steel ladles using infrared cameras and subsequent image processing for recognizing infrared thermograms on the LabVIEW platform is presented [17,18]. The results of the system’s operation in steel production are presented. It has been established that the system is very effective as a preventive maintenance program for stopping and minimizing breakouts. In [19], a review of the primary methods of analyzing images obtained during measurements with thermal imaging cameras is made. Examples of image processing techniques applied to technological diagnostics and predictive maintenance of units are included. The combined application of modern intelligent diagnostic methods, along with image processing techniques, contributes to improving analysis and diagnostic results. In [7], it is described that, as a result of thermal imaging measurements, the determination of the lateral dimensions of defects can be achieved by determining the extreme values of the temperature derivative over individual pixels. It is noted that this technique can be applied to defects with low noise levels. This requires the temperature field to be recorded before reaching the maximum value of the temperature contrast, defined as the difference between the maximum value of the hot spot in the defect area and the background temperature in the defect-free area. Obtaining images with high contrast and low noise levels requires determining the moment (time period) of maximum temperature contrast and coordinating it with the design conditions in the shop, as well as the possibility of monitoring the entire outer surface of the investigated object. The simultaneous fulfillment of all conditions is the optimal moment for thermographic monitoring. To utilize the full lifetime of the PSC, it is necessary that it be taken out for major repairs when a certain degree of wear is reached [6], which guarantees structural integrity, rather than when a certain number of operating cycles is reached. In the process of developing digital manufacturing [19], a laser scanner is used to determine the thickness of the refractory insulation, and infrared (IR) cameras are employed to monitor the temperature of the metallurgical ladle shell. Their application for monitoring an electric arc furnace (EAF) with periodic measurements from laser scanners is considered, aiming to predict the wear of the lining and optimize maintenance through shotcrete, as well as predict the service life. Applications in the RH degasser and ladle furnace (LF) are also presented. Data from IR cameras is used to detect hot spots, thereby avoiding unplanned shutdowns of the furnaces. This data is combined with data from the condition monitoring process as a tool for preventive maintenance. Both types of data are used to generate algorithms for self-learning systems as a tool for predicting and reducing unplanned downtime. Image processing and analysis algorithms are applied to support decision-making on the current condition and maintenance planning of refractory materials. The developed cyber-physical model enables decision-making without human intervention and optimizes processes for safe operation with the longest possible service life, thereby achieving the best price-to-performance ratio.

In modern non-ferrous metallurgical production, Peirce–Smith converters (PSCs) are a crucial component in the technological process, and their stable operation is essential for the overall operation of the entire technological line. This draws attention to them and refractory insulation, the condition of which must be monitored and controlled. The refractories in these units operate under severe conditions. During the technological process, high temperatures are achieved, the insulation is subjected to cyclic temperature changes, and the movement of the liquid metal, as a result of the blowing, further contributes to the erosion of the thermal insulation, along with the chemical one. In the operation, the most problematic area of converters is the tuyere row, where the refractory insulation wears out at the highest rate. During matte blowing, deposits form at the nozzle outlet, which must be removed [20]. The puncture of the nozzles during the removal of deposits, in combination with the high velocity of the injected air, turbulent flows, and the presence of molten phases in the liquid bath of the converter, can cause direct mechanical erosion of the refractory material. Excessive enrichment with oxygen can lead to higher temperatures at the nozzle outlet, thereby accelerating the erosion of the refractory material. For this purpose, the majority of the conducted research and publications [20,21,22,23,24,25,26,27] aim to optimize refractory insulation, study the hydrodynamics of the converter bath, investigate the influence of purging parameters, and maintain the tuyere section.

The developed concepts of predictive maintenance systems [28,29,30,31,32,33] are primarily based on managing the parameters of purging liquid metal and maintaining the tuyere zone and its adjacent section. In parallel with the wear of the tuyere zone, the rest of the refractory insulation that is in contact with the liquid metal and slag is also subjected to the physicochemical effects of the interaction with the liquid metal. Studies [34] have shown that 65% of the wear on the refractory insulation of the non-defective area of the wall under the tuyere zone is due to thermal cracks forming in the vertical direction, and 35% is due to chemical erosion. As a result of the chemical interaction between the liquid metal and the refractory wall, an infiltrated reaction layer with different thermomechanical properties from those of the refractory material is created on the contact surface of the refractory [35,36,37]. These weakened refractory microstructures are exposed to additional thermal and mechanical loads, leading to both continuous degradation (hot erosion and corrosion) and the formation of cracks (thermochemical cleavage), as well as further destruction. To these mechanisms of destruction of refractory materials, temperature changes, and the cyclic operation of converters can be added [37]. Temperature fluctuations during the converter’s periodic operation are sources of thermal stress in refractory materials. The latter expand and contract unevenly, which leads to the appearance of cracks and destruction. The cyclic operation of converter units, characterized by alternating periods of blowing liquid metal and standstill after draining the metal, results in thermocycling. As a result, the refractory material expands and contracts, causing the joints to open. Such destruction during the operation manifests as damage in individual zones of the wall, allowing the liquid metal to penetrate deeply into the wall, which is a prerequisite for a production accident. These defects in the process of operation develop in parallel with the wear of the tuyere zone and the general wear of the wall. This necessitates periodic monitoring and maintenance decisions that take into account local damage outside the tuyere area of the unit at various stages of its life cycle. Such investigations for infrared diagnosis of PSC are not presented in the technical and scientific publications.

In the production conditions of some metallurgical companies during operation, thermal imaging measurements of the converter surface are taken, based on which assumptions are made about the erosion of the insulation and the presence of local damage, without any additional diagnostic procedures or condition assessments. The development and introduction of evaluation procedures based on thermographic monitoring requires conducting research and developing a technological regulation and a specific approach to evaluate the current condition of the units, to extend the life cycle, and safely utilize the maximum resource. The investigation aims to establish the conditions for organizing and conducting thermographic diagnostic procedures as part of a technological regulation for thermovision diagnostics and decision-making in predictive maintenance of converter units in non-ferrous metallurgy.

This paper presents an approach for implementing periodic thermographic monitoring of a Peirce–Smith Converter (PSC). The object of the study is the refractory insulation in the part of the wall in contact with the liquid metal and slag (excluding the tuyere zone). Due to the cyclic load and the impossibility of continuously measuring the external surface temperature, it is proposed to implement periodic monitoring, taking into account the features of double-sided active thermography. For this purpose, a 3D mathematical model has been developed to account for the heat exchange processes in the wall of the region described above during the technological process. The procedure for applying the mathematical model to simulate the temperature field on the outer surface at different stages of the life cycle, taking into account local damage, is presented. The temperature field on the external surface has been studied at various stages of the PSC life cycle, including the presence of local damage. Based on the analysis of the temperature contrast, two time ranges for conducting thermographic monitoring have been determined. They meet the thermal, structural, and technological requirements. The presented approach can be adapted to implement periodic thermo-graphic monitoring of other metallurgical units with cyclic operation. Based on simulation modeling results, a regression equation has been derived to determine the thickness of the refractory layer in the defect-free area as a function of the outside surface temperature in the same area of the wall.

## 2. Methods

**Figure 1:**
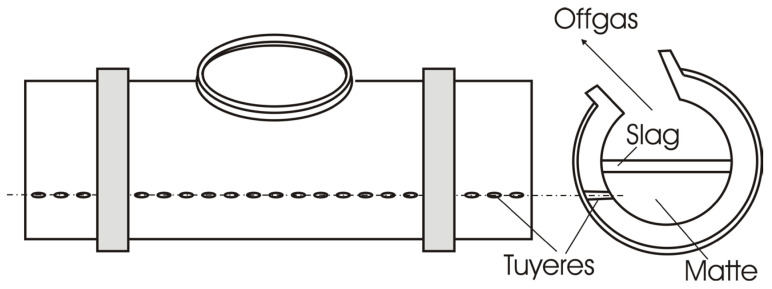
Schematic representation of a converter unit.

The object of the presented research is the Peirce–Smith converter processing copper matte in non-ferrous metallurgy, shown in Figure 1. It is a technological facility with periodic operation, consisting of a horizontal cylindrical reactor, a support device, a mechanism for rotating, a tuyere for supplying air, and a hole for removing converter gases. The oxidation air, enriched with pure oxygen, is provided to the copper matte melt through a series of tuyeres. The liquid matte is fed into the converter from the furnace with specialized metallurgical ladles and is poured into the charging hole.

When performing the individual operations of the converter unit, it is positioned in a fixed location in the workshop. During the loading and draining of the liquid metal, it rotates around its horizontal axis. For the purging process, an oxidizing agent and the necessary additives for processing are supplied, while the structure is in an upright state, and the level of the liquid metal does not exceed half the height of the working space.

In previous studies by the research team [37], the operating and technological parameters of a Peirce–Smith converter were established through an industrial experiment. Based on a statistical analysis of data from real work, the limits of variation, the duration of individual operations, and the temperature of the metal during the technological process have been determined [37]. The technological process for converting includes two periods of metal processing. The first period of converting comprises loading the first portion of copper matte, blowing, and casting the first portion of slag. This is followed by loading an additional amount of liquid metal, blowing, and casting the second portion of slag. The second period of converting consists of blowing and casting the processed metal. This is followed by the preparation of the converter for the next operation, which includes cleaning the blowing tuyeres and aperture the unit. The new technological cycle begins with loading a portion of copper matte, and the technological operations are performed again. The service life of a converter lasts between two and three months, during which time 260 to 330 technological cycles are realized, forming one campaign. From the point of view of maintaining the converters, after a certain degree of wear of the refractory insulation in the blowing zone, it must be replaced. For the sustainable operation of the converter, three intermediate repairs of the tuyere zone are provided, after which the refractory insulation of the entire unit is subject to repair.

In this study, attention is focused on the condition of the refractory insulation in the area of the unit that contacts the liquid metal during the technological process, which may prove problematic during the last stage of converter operation, after the third interim repair. To establish the possibilities for conducting infrared diagnostic procedures, it is necessary to determine the optimal period for conducting thermovision measurements from a structural and thermal engineering perspective. Based on the study’s results, further research is needed to determine a dependence for predicting the wear of the refractory converter wall.

The location of the converter units in the workshop and the organization of the technological process do not allow the application of online systems for diagnostics and monitoring of their entire surface. Therefore, the diagnostic procedures are based on periodic measurements of temperature fields using infrared cameras, employing a passive thermographic approach. The cyclical operation of the converter units and the associated transient temperature field in the refractory wall require the implementation of a combined approach, taking into account the features of active two-sided diagnostics. Basic parameters for conducting such monitoring to diagnose local damage include the temperature distribution in the hot spot of the outer surface in the defect area, its maximum value (T_m_), and the surface temperature in the defect-free region (T_f_), also known as the background temperature. The difference between the values of maximum temperature in the hot spot area and the background temperature represents the differential temperature signal, also known as the temperature contrast. Taking into account the features of two-sided active thermography in the diagnosis of local damage requires tracking the temperature contrast value over time and determining the moment τ_m_ at which it reaches its maximum value [6]. This is the final moment by which the thermographic monitoring should be implemented. In practice, the so-called “early monitoring” is also applied at a time τ < τ_m_. The advantage of early measurement is the low value of heat diffusion in the transverse direction. It is applicable for dynamic analyses, as it is characterized by an increase in surface temperature over time. When conducting thermographic measurements in the time range of maximum temperature contrast, the signal-to-noise ratio is expected to be at its maximum; i.e., the contrast between the measured temperatures in the defect area and the defect-free area is expected to be at its greatest. To conduct diagnostic procedures based on the values of the temperature contrast and other parameters, detailed information is required about the temperature field change on the surface during the technological process in the presence of local defects, which necessitates the application of simulation models. For the study, analysis, and diagnostics of surface temperature fields resulting from damage, a 3D mathematical model of the transient heat transfer in the wall of a converter unit in non-ferrous metallurgy has been developed and numerically implemented. To adjust the operation of the mathematical model, the design parameters and the results of an industrial experiment [37] were used, considering the operating and technological parameters of the production process. With the help of the model, various combinations of the wear levels of the refractory layer on the wall and the parameters of a local defect were simulated for different stages of the converter’s service life.

The non-stationary temperature distribution along the wall thickness is described using the Fourier equation in cylindrical coordinates *z, r,* and *φ*:

(1) $$\rho c\frac{\partial T}{\partial \tau }=\frac{1}{r}\frac{\partial }{\partial r}\left(k(T)r\frac{\partial T}{\partial r}\right)+\frac{1}{r^{2}}\frac{\partial }{\partial \varphi }\left(k(T)\frac{\partial T}{\partial \varphi }\right)+\frac{\partial }{\partial z}\left(k(T)\frac{\partial T}{\partial z}\right)$$

where *k* is thermal conductivity coefficient, W/mK; *T*—wall temperature, °C; *r*—radius, m; *c*—specific heat capacity, kJ/kgK; *ρ*—density, kg/m^3^; *z, r* and *φ*—coordinates; and *τ*—time, s.

With steady-state thermal conductivity, the temperature remains constant over time, and the above expression takes the form

(2) $$k\left(t\right)\left(\frac{\partial^{2}T}{\partial z^{2}}+\frac{\partial^{2}T}{\partial r^{2}}+\frac{1}{r}\frac{\partial T}{\partial r}+\frac{1}{r^{2}}\frac{\partial^{2}T}{\partial \varphi^{2}}\right)=0$$

When solving the equation, it is assumed that the liquid metal is perfectly homogeneous and stationary during the technological process, the temperatures of the metal and slag are the same, and the wear of the wall is uniform along its length. Based on the assumptions and considerations outlined above, the boundary conditions can be presented as follows.

For the inner surface of the wall in the area of close contact with the metal,

(3) $${k\left(T\right)gradT\left(z,\varphi \right)|\;}_{r=r1}=h\left(T_{L}-T_{WS}\right)$$

where *T_L_* is the temperature of the liquid metal, °C; *T_WS_*—the temperature of the inner (hot) surface of the wall, °C; *r*_1_—the radius of the inner surface of the wall, m; and *h*—the heat transfer coefficient from the metal to the wall [38], W/m^2^K.

For the inner surface of the wall in the absence of metal or the area above the level of the metal:

(4) $${k\left(T\right)gradT\left(z,\varphi \right)|\;}_{r=r1}=q_{inc}$$

where *q_inc_* is the resultant heat flux to the inner surface of the wall in the absence of metal or the area above the metal level, W/m^2^.

For the outer surface

(5) $${k\left(T\right)gradT\left(z,\varphi \right)|\;}_{r=r2}=h_{\Sigma }(T_{S}-T_{A})$$

where *r*_2_ is radius of the outer surface of the wall, m; *T_A_*—ambient temperature, °C; *T_S_*—temperature of the outer surface of the converter unit, °C; *h*_Σ_* = (h_c_ + h_r_)*—total heat transfer coefficient, W/m^2^K; *h_c_*—convective component of the heat transfer coefficient [26], W/m^2^K; *h_r_ = q_r_/(T_W_ − T_A_)*—radiant component of the heat transfer coefficient, W/m^2^K; and *q_r_*—radiation heat flux from the outer surface to the environment, W/m^2^.

To account for the temperature distribution at different stages of the converter operating cycle, it is necessary to perform calculations varying in degree of wear, i.e., at various sizes of the wall thickness and local damage. To determine the initial condition, the heat conduction Equation (1) is solved under steady-state conditions in the form represented by Equation (2) when setting a first-order boundary condition:

(6) $$\;{T(z,\varphi )|\;}_{r=r1}=T_{WS}$$

where *T_WS_* is the temperature of the internal wall surface at the end of the heating process after repair and before the start of operation of the unit.

**Figure 2:**
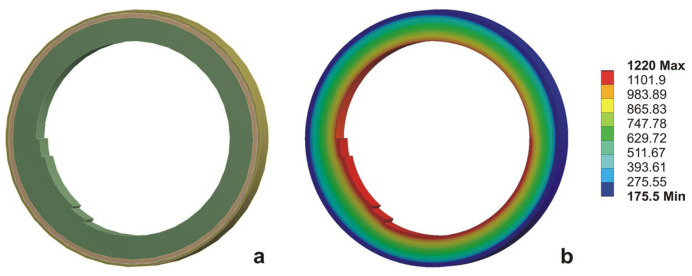
Computational object and temperature distribution on the wall, (**a**) object of investigation, (**b**) temperature distribution in the investigated object.

The obtained results for the temperature distribution from the computational procedure under steady-state conditions are assigned as the initial condition for solving the transient heat conduction equation. For the numerical implementation of the mathematical model, the finite element method and the Ansys 16 software product were used. To account for the individual stages of operation and downtime, several technological cycles were sequentially simulated, with the converter being full of liquid metal during operation and empty during downtime between two technological cycles. A ring-shaped computational object was built to conduct the study, representing a portion of the unit’s cross-section, perpendicular to the horizontal axis. The computational object, along with a visualization of the temperature distribution in the wall, is presented in Figure 2.

It is assumed that the metal is filled and poured from the unit instantly. During the operation of the converter, its lower half is full of liquid metal. When idle between two technological cycles, the unit is empty, in which case the walls are cooled by heat transfer through convection and radiation from the outer surface. Determining the surface temperature distribution for different stages of the converter’s operational period requires that the calculation procedures be carried out at various thicknesses, reflecting the degree of wear: initial thickness, thickness in the middle of the campaign, and thickness at the end of the campaign, upon which the converter must be taken out of service. When conducting the study for each calculation variant to determine the temperature distribution, the Fourier equation is solved under steady-state conditions, and the results obtained are assigned as the initial condition for studying the transient heat transfer. When realizing the calculations, the unsteady heat transfer is solved under several successive cyclic repetitions of the boundary conditions characteristic of the converter operating cycle. The determination of the actual temperature distribution in the volume at a specific wall thickness and the corresponding conditions is carried out for a fixed moment τ of the technological cycle after fulfilling the following conditions:

(7) $$\left|T_{Smax}\left(\tau ,\;n\right)-T_{Smax}\left(\tau ,\;n-1\right)\right|<\;0.3\;{}^{\circ}C$$

(8) $$\left|T_{Smin}\left(\tau ,\;n\right)-T_{Smin}\left(\tau ,\;n-1\right)\right|<\;0.3\;{}^{\circ}C$$

where n is the number of iterations performed.

The test calculations conducted show that, at constant values of the wear parameters, after simulating a certain number of consecutive cycles of the technological process, the repeatability of the values obtained for the change in the temperature field is observed. Therefore, it is accepted that the study’s results should be accounted for after fulfilling the conditions cited above in Equations (7) and (8). To more effectively utilize available computational resources, reduce calculation time, and minimize the volume of generated result files, a series of preliminary calculations, analyses, and summaries was conducted. This aimed to reduce the computational object and determine the necessary boundary conditions for conducting the simulation study. The computational procedures performed took into account the actual temperature regime of the metal, presented in Figure 2, and the operating parameters of the process. The obtained results for the temperature distribution in the wall volume, especially on the hot surface, were subjected to generalization and analysis. This allowed the establishment of a boundary condition, represented by Equation (9), at the contact areas of the liquid metal with the refractory insulation of the wall.

(9) $${\lambda \left(T\right)gradT\left(z,\varphi ,\tau \right)|\;}_{r=r1}=T_{WS}(\tau )$$

**Figure 3:**
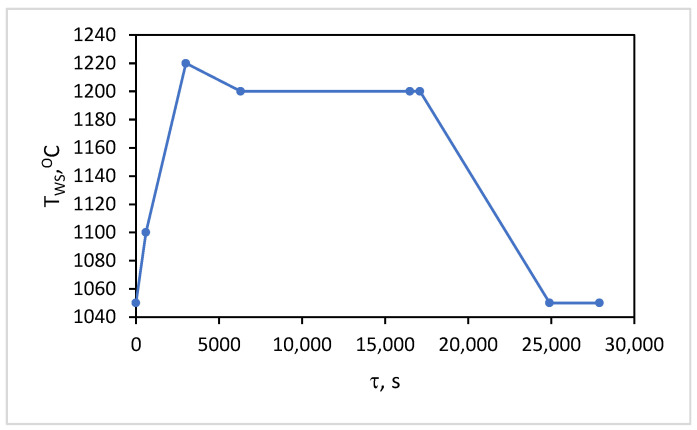
Variation in the boundary condition values *T_WS_(τ)* on the inner surface.

The change in the values of the boundary condition *T_WS_ (τ)* of the hot (internal) surface for one technological cycle is presented in Figure 3.

**Figure 4:**
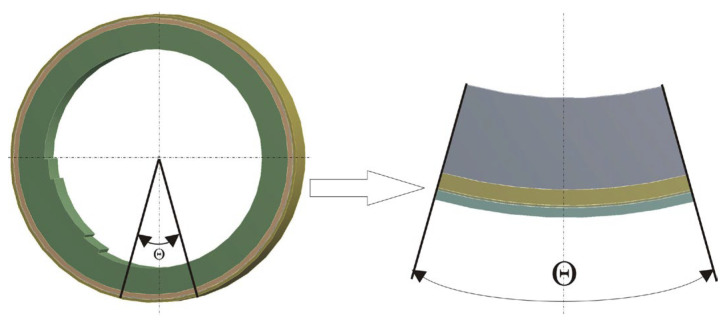
Computational object for studying heat transfer in the wall of the converter unit.

While applying this boundary condition and taking into account the symmetry along the horizontal axis of the converter and the need to study the area in contact with the liquid metal, to obtain results for the temperature on the outer surface of the wall, it is accepted that the computational object could be reduced and represented by a sector of the wall with a central angle θ, presented in Figure 4.

The described mathematical model, with the formulated initial and boundary conditions, was used to study the temperature field on the surface of the converter unit wall, depending on the parameters of local damage and the wear of the working refractory layer. The numerical realization of the mathematical model is presented in Appendix A.

## 3. Results and Discussion

In the practice of the predictive maintenance of the PSC, in addition to the condition of the tuyere zone, the presence of local defects and the wear-thinning (wear-induced thinning of the layers) in the rest area of the wall refractory lining in contact with the liquid metal should be considered and evaluated. The development of the wear-thinning process depends on the chemical composition and temperature of the liquid metal and slag, the movement of turbulent metal flows, etc., as described in Part 1, Introduction. This wear leads to a gradual thinning of the refractory lining after the converter is put into operation and continues throughout its entire life cycle. If we assume that wear occurs evenly across the entire contact zone between the liquid metal and slag with the refractory insulation, then the temperature of the outer surface of the wall will be close to T_f_. These values are characteristic in the absence of local damage. In the following discussions, the defect-free zone will be referred to as the uniform-wear zone. During operation, the thinning of the refractory layer leads to an increase in the temperature of the outer surface, due to a decrease in the thermal resistance of the wall. This process occurs over the entire contact surface, regardless of the presence of local defects, which may be absent. Thinning the thickness of the refractory insulation in the defect-free area beyond a particular value leads to an excessive increase in the surface temperature, which is reflected in the strength state of the outer PSC steel structure. At the end of the life cycle, the thickness of the refractory layer approaches the critical value that ensures the converter’s trouble-free operation.

The presence and development of a local defect cause the formation of a hot spot on the outer surface of the converter. The appearance of this damage is caused by mechanical or thermal stresses. The deeper the defect, the thinner the residual thickness of the refractory wall in front of the defect, and the higher the temperature in the hot spot on the outer surface. The temperature distribution in the hot spot is characterized by a maximum T_m_, and it gradually decreases in value until it reaches the background temperature T_f_. The last is known as the temperature of the surrounding, uniform-wear zone. The maximum surface temperature T_m_ is the highest temperature observed in the area of the localized defect. When the depth of the defect reaches the thermal insulation layer, it is destroyed, and a breakout and metal leak can occur. For this reason, the PSC must be taken out of service before the residual thickness of the refractory layer reaches a certain critical value. Thus, in both cases of wear-induced thinning and local damage, there are critical thickness values for the refractory layer. At these values, the converters must be shut down and repaired before a failure occurs.

Local damage can occur and begin to develop at different stages of the converter’s life cycle. Thus, at the different stages of the life cycle, both types or only the wear-induced thinning can be observed in the converter wall. That is why two areas can be conditionally formulated in the refractory insulation [6,7]. The first is a uniform-wear zone, characterized by an external surface temperature T_f_. The second is a zone of local defect (referred to as the localized wear zone below in the text), characterized by the presence of a hot spot on the outside and a maximum temperature, T_m_.

The difference between the maximum temperature T_m_ in the hot spot of the defect area and the surface temperature in the uniform-wear area T_f_ represents the temperature contrast T_c_, which is used to assess the severity of the local defect. Analysis of its behavior over time helps to determine the extent of damage and the optimal time for inspection. The maximum value of the temperature contrast over time is a critical point for thermographic analysis. The time at which the temperature contrast reaches its peak indicates the optimal time for thermographic analysis. This is the point at which the signal-to-noise ratio is expected to be highest, meaning that the difference between the localized wear area and the uniform-wear area is most pronounced. Taking measurements before the temperature contrast reaches its maximum is known as early monitoring. It is applied for dynamic analysis [6].

To study the heat transfer processes in the converter wall, a local damage with the shape of a regular prism was simulated in the developed 3D mathematical model, resembling a defect resulting from a destroyed brick from the refractory (magnesia-chromite) layer of the aggregate. The wall of the converter consists of a steel structure with a thickness of 0.04 m, a compensation layer of fireclay powder with a thickness of 0.01 m, a layer of fireclay bricks with a thickness of 0.064 m, and an inner layer of magnesia-chromite material with a thickness of 0.375 m. In the following discussion, both fireclay layers will be referred to as the thermal insulation layer, and the inner one, made of magnesium-chromite material, will be referred to as the refractory layer.

**Figure 5:**
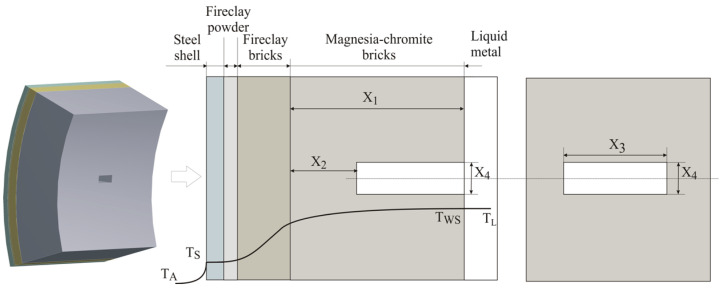
Schematic representation of the converter wall, defect, and accepted designations.

A schematic representation of the defect configuration and the adopted designations is visualized in Figure 5. The following designations of the geometric characteristics have been adopted to represent the damage parameters:

- The thickness of the wear-thinning refractory lining of the wall X_1_ in the uniform-wear zone;
- The residual thickness of the refractory lining of the wall X_2_ in the localized wear zone;
- Length of the fault X_3_;
- Width (height) of the damage X_4_.

For the study, it was assumed that the width (height) of the defect on the inner surface of the wall was constant and of size X_4_ = 0.06 m, and the remaining three parameters were varied at three levels: minimum (−1), average (0), and maximum (+1). A complete factorial experiment was planned and implemented, requiring 27 experiments. When designing the experiment, the values of the thickness of the refractory (magnesia-chromite) layer in the uniform-wear zone (X_1_), the residual thickness of the insulation in front of the defect in the localized wear zone (X_2_), and the length of the defect (X_3_) are varied according to the values presented in Table 1. Levels (−1) with a value of 0.195 m for X_1_ and 0.005 m for X_2_ are the critical values, at which the converter must be taken out of service and repaired.

The experimental plan, including the levels and values of the parameters, is presented in Table 2.

**Figure 6:**
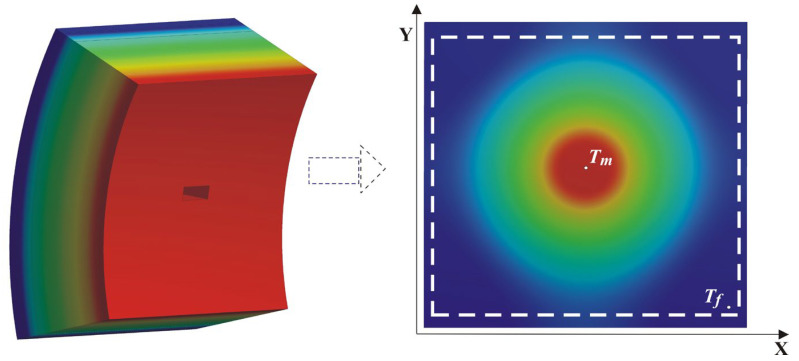
Temperature distribution in the wall and on the outer surface in the presence of a local defect.

To conduct the simulation calculations, the described mathematical model was numerically implemented, with the computational procedures being carried out for a section of the wall presented in Figure 4. A visualization of the obtained results for the surface temperature field is presented in Figure 6. The figure shows that, as a result of the heat exchange processes, the presence of local damage in the converter wall is projected onto the surface temperature field as a hot spot.

**Figure 7:**
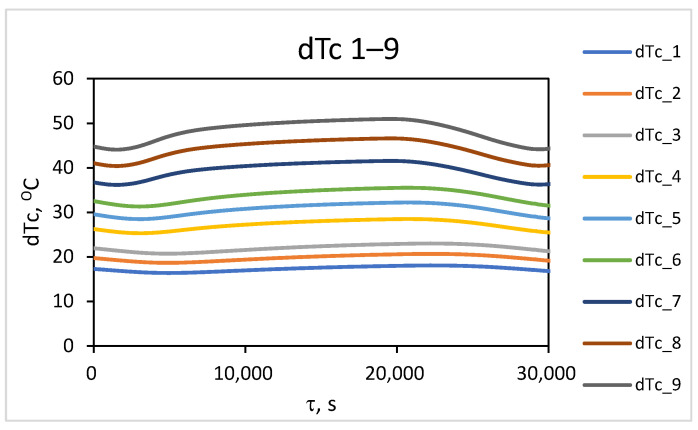
Change in temperature contrast at the beginning of the life cycle at X_1_ = 0.375 m.

**Figure 8:**
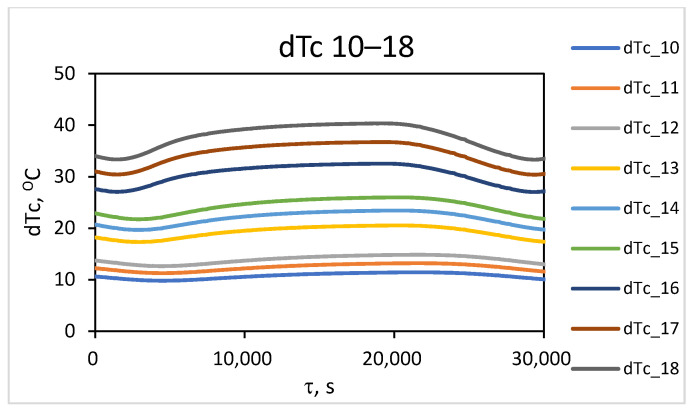
Change in temperature contrast at the middle of the life cycle at X_1_ = 0.285 m.

**Figure 9:**
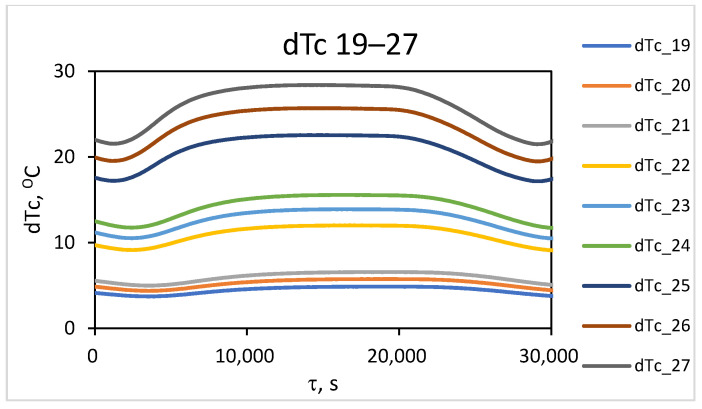
Change in temperature contrast at the end of the life cycle at X_1_ = 0.195 m.

From the simulation study results, the temperature distribution along the outer surface of the wall during the converter operating cycle was accounted for. To investigate the temperature contrast, the change in maximum temperature (T_m_) in the localized wear zone and the temperature (T_f_) in the uniform-wear zone, as presented in Figure 6, is examined. The difference between the values of these temperatures over time represents the change in temperature contrast throughout the converter’s operating cycle, dT^τ^_C_ = T^τ^_m_ − T^τ^_f_. The results obtained for the change in temperature contrast dT^τ^_C_ (denoted as dT_C_) throughout one operating cycle at different stages of the operational period (campaign) are presented in Figure 7, Figure 8 and Figure 9. Figure 7 presents the results of the studies for the first nine combinations of the experimental design (Table 2), relating to the initial wall thickness X_1_ = 0.375 m, i.e., at the beginning of the refractory insulation life cycle, in the absence of wear. Figure 8 presents the results of the studies for the second nine combinations of the experimental design, relating to refractory insulation thickness in the uniform-wear zone (X_1_ = 0.285 m), i.e., in the middle of the campaign (life cycle). Figure 9 presents the results of the studies for the third nine combinations of the experimental design, relating to the wall thickness in the uniform-wear zone X_1_ = 0.195 m, i.e., at the end of the refractory insulation campaign.

From Figure 7, Figure 8 and Figure 9, it can be seen that during the technological cycle, the temperature contrast passes through minimum and maximum values. Figure 7 shows that for combination No. 1 from Table 2 of the experimental plan, the minimum value of the temperature contrast is observed at 5000 s from the beginning of the technological cycle, while the maximum is for combination No. 9, at 19,700 s. A summary of the times at which the minimum and maximum values of the temperature contrast are observed for the three levels of thickness of the refractory wall in the defect-free area is presented in Table 3.

Table 3 shows that the compromise zone between the maximal and minimal values for all combinations can be formulated as the range between the highest value of the minimum levels and the lowest value of the maximum levels. They are accounted for by the minimal value of the change in temperature contrast for combination No. 1 and the maximal value for combination No. 27, as presented in the experimental plan in Table 2. It can be summarized that the zone in which the time for conducting the thermographic control should be selected is within the range of 5000–17,200 s from the beginning of the technological cycle. Considering the design and technological features of the process implementation, three time intervals are suitable for conducting measurements across the entire surface. These are the times for pouring the slag after the first and second stages of blowing, as well as the time for pouring the black copper at the end of the process. In these stages, the converter is turned around its horizontal axis, allowing for thermographic measurements of the lower part of the cylindrical body’s surface. The time for pouring the slag after the second blowing, at the end of the first period (during the interval of 5700 ÷ 6300 s in Figure 9), is suitable for conducting measurements for early diagnostics. The time of pouring the black copper at the end of the process (with an interval of 16,500 ÷ 17,100 s, Figure 9) is suitable for diagnostics at maximum temperature contrast. The results obtained for the transient temperature field on the external surface of the converter for the different combinations of the planned experiment enable the background temperature values to be accounted for at the beginning of the two time intervals, which are suitable for thermographic monitoring.

**Figure 10:**
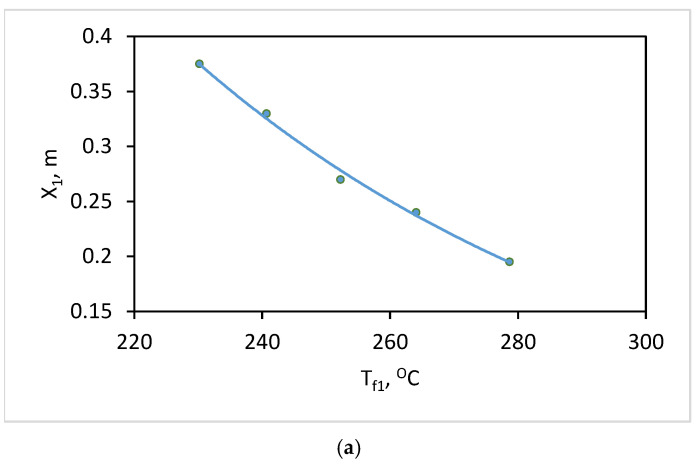

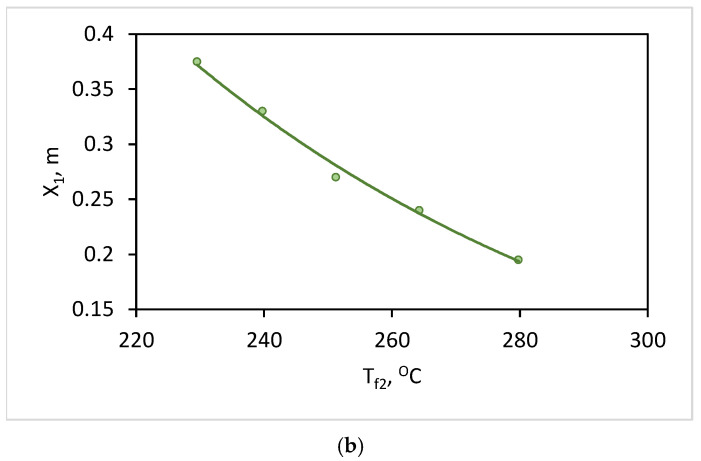
Graphical dependence between the values of the background temperature (T_f_) in the uniform-wear zone and the corresponding thicknesses of the refractory insulation (X_1_): (**a**) for the time range of slag draining—blue color; (**b**) for the time range of the liquid metal pouring—green color.

In the experimental plan, the level (+1) for X_1_ has a value of 0.375 m, representing the initial wall thickness after commissioning. Level (−1) with a value of 0.195 m is the critical value at the end of the life cycle, at which the converter must be taken out of service and repaired. The average level (0) has a value of 0.285 m and can be assumed to be in the middle of the life cycle. In predictive maintenance, the units are taken out of service not after a certain number of cycles, but based on an assessment of their actual condition. Using the developed mathematical model, the surface temperature distribution during the technological cycle was determined when setting the values of the thickness in the uniform-wear zone X_1_ at five levels, as follows: (−1), (−0.5), (0), (+0.5), (+1). From the obtained results, the values of the temperature T_f_ in the defect-free area are accounted for each of the two time ranges of the study. The obtained results are presented graphically in Figure 10a (for the time range of slag draining) and Figure 10b (for the time range of the liquid metal pouring). In these figures, T_f1_ refers to the background temperatures during the slag tapping period, and T_f2_ refers to the period at the end of the technological process. In this way, for specific values of T_f_ in the uniform-wear zone (determined within the time intervals for thermographic inspection), the thickness of the refractory insulation in this zone can be established, regardless of the number of technological cycles.

The results for the background temperature and the corresponding refractory insulation thickness for the two measurement periods were subjected to a correlation analysis, the results of which are presented in Table 4.

From the obtained values in the correlation matrix, it can be seen that between the background temperature T_f_ and the wall thickness in the uniform-wear zone for both cases of study, the correlation coefficient has values R > 0.98. This allows the background temperature to be considered as a diagnostic parameter, based on which a relationship can be established to assess the thickness of the refractory layer in the uniform-wear zone of the wall. As a result of statistical processing of the results, regression dependencies have been derived, allowing for the prediction of wall thickness in the uniform-wear zone based on the background temperature of the outer surface, as represented by Equations (10) and (11):

(10) $$X_{1}=8.3635e^{-0.013.Tf1}$$

(11) $$X_{1}=7.2998e^{-0.013.Tf2}$$

The graphical dependencies are presented in Figure 10a,b. The multiple correlation coefficients for both regression dependencies are R^2^ = 0.995 for the early period of inspection and R^2^ = 0.993 for the period of maximum temperature contrast.

For the relevant time ranges, further research will seek to identify diagnostic features and dependencies for assessing the parameters of the local defect and developing a technological regulation for diagnosing the condition of the refractory insulation as part of predictive maintenance procedures.

## 4. Conclusions

In the investigation, an approach for implementing thermographic monitoring of a Peirce–Smith converter is presented. The implementation of periodic monitoring is proposed, taking into account the specifics of bilateral active thermography. To determine the optimal period for conducting thermographic monitoring of the wall of a converter unit, the change in the surface temperature field in the presence of local damage to the internal surface has been investigated. The behavior of the temperature contrast during the technological process of the converter and at different stages of the operating cycle was analysed. It was found that from a technological, constructive, and thermal engineering point of view, the time at the end of the first period when pouring the slag after the second blowdown can be used for early diagnostics, and the time of pouring the liquid metal after the second period can be used for diagnostics at a maximum differential temperature signal. Based on correlation analysis, it was established that background temperature can be used as a diagnostic parameter. Statistical processing of the results for the surface temperature distribution values established regression dependences, allowing the determination of the wall thickness as a function of surface temperature in the uniform-wear zone (defect-free area).

The established periods will be utilized in future research to develop diagnostic procedures for assessing the parameters of the damage and the actual condition of refractory insulation when implementing predictive maintenance procedures for converter units in non-ferrous metallurgy. The presented approach can be adapted to implement periodic thermo-graphic monitoring of other metallurgical units with cyclic operation.

## Figures and Tables

**Table 1:** Levels of amendment on the parameters of the defect.

Level	X_1_	X_2_	X_3_
+1	0.375	0.105	0.150
0	0.285	0.055	0.200
−1	0.195	0.005	0.250

**Table 2:** Experimental plan.

No.	X_1_		X_2_		X_3_	
	Level	Value, m	Level	Value, m	Level	Value, m
1	1	0.375	1	0.105	1	0.150
2	1	0.375	1	0.105	0	0.200
3	1	0.375	1	0.105	−1	0.250
4	1	0.375	0	0.055	1	0.150
5	1	0.375	0	0.055	0	0.200
6	1	0.375	0	0.055	−1	0.250
7	1	0.375	−1	0.005	1	0.150
8	1	0.375	−1	0.005	0	0.200
9	1	0.375	−1	0.005	−1	0.250
10	0	0.285	1	0.105	1	0.150
11	0	0.285	1	0.105	0	0.200
12	0	0.285	1	0.105	−1	0.250
13	0	0.285	0	0.055	1	0.150
14	0	0.285	0	0.055	0	0.200
15	0	0.285	0	0.055	−1	0.250
16	0	0.285	−1	0.005	1	0.150
17	0	0.285	−1	0.005	0	0.200
18	0	0.285	−1	0.005	−1	0.250
19	−1	0.195	1	0.105	1	0.150
20	−1	0.195	1	0.105	0	0.200
21	−1	0.195	1	0.105	−1	0.250
22	−1	0.195	0	0.055	1	0.150
23	−1	0.195	0	0.055	0	0.200
24	−1	0.195	0	0.055	−1	0.250
25	−1	0.195	−1	0.005	1	0.150
26	−1	0.195	−1	0.005	0	0.200
27	−1	0.195	−1	0.005	−1	0.250

**Table 3:** Times at which the minimum and maximum values of the temperature contrast are observed.

Combination	Time, s	
	dTc Min	dTc Max
1–9	5000	19,700
10–18	4600	18,700
19–27	3495	17,200

**Table 4:** Correlation matrix between background temperatures and their corresponding refractory insulation thickness.

	T_f1_	T_f2_	X_1_
T_f1_	1		−0.99118
T_f2_	0.999697	1	0.98777
X_1_	−0.99118	0.98777	1

## Data Availability

The original contributions presented in this study are included in the article. Further inquiries can be directed to the corresponding author.

## Appendix A

In the numerical implementation of the 3D mathematical model, the Finite difference method was used. The “Mechanical Physics Preference” option is applied to construct the computational mesh. Quadrilateral elements were selected for the metal structure of the outer surface of the PSC. As a boundary condition on the internal surface is applied, established by preliminary calculations, a temperature variation over time, represented by Equation (9), and shown in Figure 3. The boundary condition for the outer surface accounts for heat flow due to radiation and convection, as represented in Equation (5) of the mathematical model. The actual design parameters of the wall of a working PSC, the times of individual operations, and the variation in liquid metal temperature during the technological process were utilized. The thermophysical parameters of the magnesia-chromite refractory are presented in Table A1. In Table A1, the thermal conductivity coefficient and density are obtained from the manufacturer’s data (rhimagnesita.com).

**Table A1:** Thermophysical parameters of magnesia-chromite refractory material [39,40].

T, °C	k, W/(m.K)	c, J/(kg.K)	ρ, kg/m^3^
500	2.70		
750	2.60	800	3210
1000	2.60		
1200	2.60		

Thermophysical parameters of fireclay refractory material and structural steel are presented in Table A2 and Table A3.

**Table A2:** Thermophysical parameters of fireclay refractory material [39].

T, °C	k, W/(m.K)	c, J/(kg.K)	ρ, kg/m^3^
500	0.88	2.70	
750	1.051	2.60	1850
1000	1.110	2.60	
1200	1.156	2.60	

**Table A3:** Thermophysical parameters of structural steel [41].

k, W/(m.K)	c, J/(kg.K)	ρ, kg/m^3^
60.5	434	7850

As a result of preliminary calculations, it has been established that obtaining a final solution requires more than 24 h of computational time, and the resulting files exceed 30 GB. This necessitated optimization of the simulation model parameters.

Considering the characteristics of the thermographic camera available for such studies at the moment, PS 800 (1024 × 768 pixels, wavelength range 7.5 to 14 μm, focal length 28 mm, field of view 25° × 19°, measurement range: −40 °C to 150 °C, 100 °C to 800 °C, 700 °C to 2000 °C, measurement accuracy ±1 °C or ±1%, whichever is greater), it has been established that the pixel size in thermograms used for industrial inspections can range from 0.015 to 0.02 m. Therefore, when choosing the dimensions of the computational grid, an Element size of 0.02 m was adopted. The time step was selected from within the range of 30 s to 120 s.

For the sensitivity analysis of the solution to the discretization parameters, a study was conducted with three values of the cell size and three values of the time step. For this purpose, the sizes of the discretization steps in space were selected as 0.01 m, 0.015 m, and 0.02 m, and for discretization in time, 60 s, 120 s, and 180 s. A developed mathematical model was used to conduct the study. The results obtained for the surface temperature values are presented in Table A4.

**Table A4:** Surface temperature values for discretization parameter analysis.

Maximum Time Step, s		Element Size, m	
	0.01 m	0.015 m	0.02 m
60	288.46	288.48	288.48
120	288.5	288.52	288.54
180	288.55	288.58	288.59

The presented results show that within the studied range of change in the discretization parameters, the maximum value of the temperature variation is 0.011 °C. This provides us with reason to accept that the values for the maximum time step (120 s) and the element size (0.02 m) can be used as discretization parameters in the investigation process. In addition, this value is lower than the measurement accuracy of the IR camera (±1 °C or ±1%, whichever is greater).
